# Paracetamol pharmacokinetics and metabolism in young women

**DOI:** 10.1186/s12871-015-0144-3

**Published:** 2015-11-13

**Authors:** Karel Allegaert, Mariska Y. Peeters, Bjorn Beleyn, Anne Smits, Aida Kulo, Kristel van Calsteren, Jan Deprest, Jan de Hoon, Catherijne A. J. Knibbe

**Affiliations:** 1 NICU, University Hospitals Leuven, Herestraat 49, 3000 Leuven, Belgium; 2 Department of Development and Regeneration, Cluster Organ Systems, KU Leuven, Leuven, Belgium; 3 Department of Clinical Pharmacy, St Antonius hospital, Nieuwegein, The Netherlands; 4 Department of Pharmaceutical and Pharmacological Sciences, KU Leuven, Leuven, Belgium; 5 Center for Clinical Pharmacology, University Hospitals Leuven, Leuven, Belgium; 6 Institute of Pharmacology, Clinical Pharmacology and Toxicology, Faculty of Medicine, University of Sarajevo, Sarajevo, Bosnia and Herzegovina; 7 Obstetrics and Gynecology, University Hospitals Leuven, Leuven, Belgium; 8 Leiden Academic Centre for Drug Research, Leiden University, Leiden, The Netherlands

**Keywords:** Acetaminophen, Glucuronidation, Oestradiol, Oral contraceptives, Paracetamol, Pregnancy, Progesterone

## Abstract

**Background:**

There is relevant between individual variability in paracetamol clearance in young women. In this pooled study, we focused on the population pharmacokinetic profile of intravenous paracetamol metabolism and its covariates in young women.

**Methods:**

Population PK parameters using non-linear mixed effect modelling were estimated in a pooled dataset of plasma and urine PK studies in 69 young women [47 at delivery, 8/47 again 10–15 weeks after delivery (early postpartum), and 7/8 again 1 year after delivery (late postpartum), 22 healthy female volunteers with or without oral contraceptives].

**Results:**

Population PK parameters were estimated based on 815 plasma samples and 101 urine collections. Compared to healthy female volunteers (reference group) not on oral contraceptives, being at delivery was the most significant covariate for clearance to paracetamol glucuronide (Factor = 2.03), while women in early postpartum had decreased paracetamol glucuronidation clearance (Factor = 0.55). Women on contraceptives showed increased paracetamol glucuronidation clearance (Factor = 1.46). The oestradiol level did not further affect this model. Being at delivery did not prove significant for clearance to paracetamol sulphate, but was higher in pregnant women who delivered preterm (<37 weeks, Factor = 1.34) compared to term delivery and non-pregnant women. Finally, clearance of unchanged paracetamol was dependent on urine flow rate.

**Conclusions:**

Compared to healthy female volunteers not on oral contraceptives, urine paracetamol glucuronidation elimination in young women is affected by pregnancy (higher), early postpartum (lower) or exposure to oral contraceptives (higher), resulting in at least a two fold variability in paracetamol clearance in young women.

## Background

Characterizing pharmacokinetics (PK) and pharmacodynamics (PD) in specific subpopulations is essential to improve therapeutic effectiveness while minimizing adverse events [1, 2]. Gender related differences in body weight, physiology (e.g. pregnancy) or endocrinology (e.g. menstrual cycle) may affect PK. This concern is also reflected in the Food and Drug Administration (FDA) guidance on bioavailability and bioequivalence studies. This guidance recommends that in vivo bioequivalence studies should be conducted in *representative* individuals, taking into account age, gender or race. If the drug is intended for use in both sexes, one should attempt to include similar proportions of male and female volunteers [3]. The same case can be built for specific settings, like drugs intended to be used during pregnancy (e.g. pruritus of pregnancy, tocolytics, gestational diabetes or hypertension) [4]. We aim to quantify the impact of covariates on paracetamol metabolism in young women, including pregnancy and postpartum [4–10].

Paracetamol is almost exclusively metabolized by the liver. In adults, only 1–4 % is excreted in urine as unchanged paracetamol while the majority is excreted as paracetamol-glucuronide (47–62 %) or paracetamol-sulphate (25–36 %) [5–10]. A smaller part (8–10 %) is oxidized by cytochrome P450 (including CYP2E1, but also CYP1A2 and possibly CYP3A4) into 3-hydroxy-paracetamol and the toxic metabolite N-acetyl-p-benzoquinone-imine (NAPQI) [5–9]. Compared to early postpartum (10–15 weeks) observations, paracetamol clearance was significantly higher (21.1 *vs* 11.7 l.h^−1^, + 80 %) at delivery. This higher clearance was due to a disproportional increase in glucuronidation (11.6 *vs* 4.76 l.h^−1^, + 144 %), a proportional increase in oxidation clearance (4.95 vs 2.77 l.h^−1^, 78 %) and primary renal clearance (1.15 *vs* 0.75 l.h^−1^, 53 %) [6]. This increase in glucuronidation clearance may in part be driven by oestradiol, and may explain within and between individual differences in paracetamol metabolism (e.g. oral contraceptives, follicular *vs* luteal phase, postpartum, pregnancy, or duration of pregnancy) in young women [6, 8, 9, 11–14]. Based on a pooled analysis, we aimed to further explore the impact of these covariates on paracetamol metabolism based on plasma and urine collections in women at delivery, in postpartum (early, or late) and healthy volunteers, either or not on oral contraceptives (OC) following intravenous (iv) paracetamol administration [6, 11, 15].

## Methods

### Study populations and design

#### Young women at delivery, in early and late postpartum

This was an open-label, 3-period PK study (at delivery, early, and late postpartum) conducted from August 2010 to March 2013 (EudraCT Number 2010-020164-37) [6, 11, 16]. The study documents (study protocol, informed consent, subsequent amendments) were reviewed and approved by the local Ethics Committee of the University Hospitals Leuven. This study followed GCP (Good Clinical Practice) and local regulations. Written informed consent of each woman (at least 18 years, adulthood according to the Belgian law) was obtained before study initiation. The study was registered (www.clinicaltrials.gov, 19 October 2015, NCT02590900).

The administration of iv paracetamol (vial containing 1000 mg in 100 ml infusion solution, Perfusalgan®, Bristol Myers Squibb Braine l’Alleud, Belgium) is part of routine multimodal analgesia following caesarean delivery in the University Hospitals Leuven [6, 11, 16]. Consequently, patient consent was restricted to the collection of additional blood samples, urine collection and the inclusion in a database (demographic and clinical characteristics). Pregnant women scheduled for elective or (semi)urgent caesarean delivery and immediate postoperative iv paracetamol pain relief were considered. Women with known paracetamol intolerance or who were already receiving paracetamol in the period of 48 h prior to study were not included [6, 11, 16].

In the first study period, an initial iv 2 g loading dose of paracetamol (two vials) was administered to the patient by the attending anesthesiologist within 5 min following delivery of the newborn. Subsequent 1 g maintenance doses were administered by the nurse at 6 h intervals for maximal 24 h with a subsequent switch to oral paracetamol. Paracetamol was administered either as 20 min (loading dose) or 10 min (maintenance dose) infusion, through a peripherally inserted venous catheter [6, 11, 16]. To further enrich the variability in clinical characteristics at delivery compared to the earlier reported dataset [16], an additional cohort of women undergoing preterm caesarean delivery was recruited.

During the second study period, a subgroup of eight women initially included in the first study period at delivery were admitted again for a single iv 2 g loading dose administration and 6 h follow up, scheduled 10–15 weeks after delivery of the newborn (early postpartum) [16]. Finally, the same subgroup of eight women were admitted again about 1 year after delivery (late postpartum), using the same study design.

For the duration of the first study period, subjects were hospitalized at the maternity ward and for the second and third study period at the Centre for Clinical Pharmacology, University Hospitals Leuven, Leuven, Belgium. Only cases with both plasma and urine observations were recruited in this cohort. At the different time points, clinical characteristics, including body weight and height, duration of pregnancy, medical conditions and the use of oral contraceptives - when applicable - were registered.

#### Young healthy, non-pregnant female volunteers

To compare observations at delivery and in postpartum with a reference group, eight healthy young non-pregnant, female volunteers (18–40 years) were recruited. Using the same sampling strategy, these women received a single iv 2 g loading dose and 6 h follow up. Clinical characteristics, including body weight and height were collected. The non-use of oral contraceptives was an explicit inclusion criterion. This was to enable comparison with another cohort of young women (*n* = 14) exposed to the same loading dose (2 g iv paracetamol), followed by 1 g q6h for 48 h published by Gregoire et al. [15]. All these women were on contraceptives, of whom 13 were on oral contraceptives (ethinyloestradiol containing pill), one used a levonorgestrel containing intrauterine device (this volunteer was classified as not exposed to ethinyloestradiol-containing oral contraceptives).

### Blood sampling and urine collection

Following delivery, seven blood samples (2 ml per sample) were collected per subject. The first three samples were collected at 1, 2 and 4 h after initiation of the 2 g loading dose. The next four samples were collected just before the next maintenance doses (i.e. at 6, 12, 18 and 24 h). Blood samples, drawn through a second, peripherally inserted venous catheter dedicated for blood sampling only, were collected into plastic lithium heparin tubes, immediately centrifuged and plasma was stored at −20 °C until analysis. In women undergoing a caesarean delivery, urine was collected through a bladder catheter. Before the first dose, the urine collection bag was emptied and a blank urine sample was collected in order to exclude the possibility of paracetamol being present in urine. Second and third urine collections were harvested from 0 to 6 and 6–24 h respectively, after the total urine volume was measured. After collection, urine samples were immediately stored at −20 °C until analysis.

In the single dose studies (early postpartum, late postpartum and healthy volunteers), a 2 g loading dose was administered to the subjects after they had voided. Four blood samples at predetermined time points (1, 2, 4 and 6 h after initiation of dosing) and one urine sample (extracted from 0 to 6 h urine collection) were collected following the same principles described for the first study period [6, 11, 16]. In the Gregoire et al. study, only plasma samples were collected during repeated intravenous paracetamol administration [15].

### Bioanalytical methods

Concentrations of unchanged paracetamol (plasma, urine) and its metabolites paracetamol-glucuronide (urine) and paracetamol-sulphate (urine) were determined by high performance liquid chromatography (HPLC), according to a previously validated and reported method [16]. The lower limit of quantification for paracetamol in plasma was 0.08 mg l^−1^, and for paracetamol and its metabolites in urine 1 mg l^−1^. Coefficients of variation for intra- and interday precision and accuracy were all below 15 % [16].

In the study of Gregoire et al., a HPLC method with UV detection was used to quantify paracetamol concentrations in plasma, following a systematic dilution procedure (max 1/20). The analytical procedure in plasma was shown to be linear from 0.020 to 10.0 mg/ml with the limit of quantification at 0.020 mg/ml [15].

Oestradiol and progesterone levels were determined for each patient at each study point via competitive enzyme-linked immunosorbent assay (ELISA) with electrochemiluminescence (MODULAR® ANALYTICS E-170, Roche/Hitachi) by the clinical laboratory of the University Hospitals Leuven [11].

### Data analysis and population PK parameter estimates

The analysis was performed using non-linear mixed effect modeling (NONMEM, GloboMax LLC, Hanover, MD, version VI) by use of the first-order conditional estimation (Method 1) with η-ε interaction and ADVAN6 TOL5. Parent drug and metabolites were modelled simultaneously. For this purpose, the amounts of unchanged paracetamol, paracetamol-glucuronide and paracetamol-sulphate excreted in urine were calculated by urinary concentration (mg l^−1^) multiplied by urine volume and subsequently converted to milligram paracetamol equivalents using a molecular weight of 151.2 mg mmol^−1^ for paracetamol, 328.3 mg mmol^−1^ for paracetamol-glucuronide and 230.2 mg mmol^−1^ for paracetamol-sulphate.

S-plus (Insightful software, Seattle, WA, version 6.2) was used to visualize the data. Model building was performed in four different steps: (i) selection of the structural model (one, two or three compartment model), (ii) choice of a statistical sub-model, (iii) covariate analysis, and (iv) model evaluation. Discrimination between different models was made by comparison of the objective function. A value of *P* < 0.01, representing a decrease of 6.63 points in the objective function, was considered statistically significant. In addition, goodness of fit plots including observations *vs* individual predictions, observations *vs* population predictions, conditional weighted residuals *vs* time and conditional weighted residuals *vs* population predictions were used for diagnostic purposes. Furthermore, the confidence interval of the parameter estimates, the correlation matrix and visual improvement of the individual plots were used to evaluate the model.

**Fig. 1:**
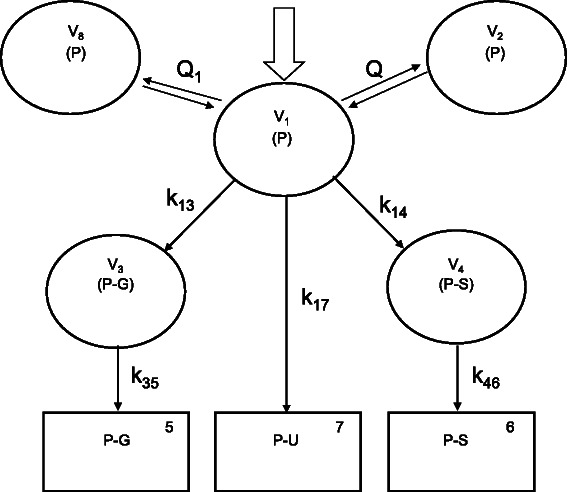
Schematic representation of the pharmacokinetic model and its metabolites in plasma and urine [Abbreviations: P, paracetamol; P-G, paracetamol-glucuronide; P-S, paracetamol-sulphate; P-U, unchanged paracetamol; V1, volume of the central compartment; V2 and V8, volumes of the peripheral compartment; Q and Q1, inter-compartmental clearances between central and peripheral compartment; k, elimination coefficients]

The paracetamol data were best described with a three-compartment model, parameterized in terms of the volume of the central compartment (V_1_), inter-compartmental clearances between central and peripheral volumes (Q and Q_1_), peripheral volumes (V_2_ and V_8_), clearance to paracetamol-glucuronide (CL_P-G_ = V_1_ * k_13_), clearance to paracetamol-sulphate (CL_P-S_ = V_1_ * k_14_), clearance of unchanged paracetamol (CL_P-U_ = V_1_ * k_17_) (Fig. 1). Clearance attributable to pathways other than these measured in urine, the oxidative metabolites (CL_P-O_) could not be significantly identified. With the current study design, the metabolite volumes of distribution of paracetamol-glucuronide and paracetamol-sulphate (V_3_ and V_4_) cannot be identified, but were fixed to 18 % of the central distribution volume of paracetamol in plasma [17]. Using this approach, the elimination rate of paracetamol-glucuronide from plasma to urine (k_35_) equals the elimination rate of paracetamol-sulphate (k_46_). Relating the rate of elimination of unchanged paracetamol (k_17_) to k_35_ and k_46_ by estimation of a multiplication factor (MF) as k_35_ = MF*k_17_ resulted in a significant decrease of objective function (ΔOF 40.9).

The uncertainty in the population parameters (coefficient of variation, CV) was estimated in NONMEM by the covariance step. Individual estimates of the PK parameters were assumed to follow a log-normal distribution. Therefore, an exponential distribution model was used to account for between individual variability. Residual errors were best described with a proportional error model. The residual error for the paracetamol data of Gregoire et al. [15] were best described with a combined additive and proportional error model.

### Covariate analysis

The covariates body weight, body height, body surface area, age, gestational age (GA), being at delivery, in early postpartum (10–15 weeks after delivery), late postpartum (1 year after delivery), healthy female volunteer, term/preterm delivery (dichotomous), twin pregnancy, maternal morbidity (pre-eclampsia, diabetes mellitus, either type 1 or gestational), use of oral contraceptives, oestradiol and progesterone levels and urine production (ml h^−1^) were plotted subsequently against the individual post-hoc parameter estimates and the weighted residuals to visualize potential relationships. Based on these plots, covariates were tested for their influence. Starting from the basic model without covariates, the covariate model was first built up using forward inclusion (*p* < 0.005, representing a decrease of 7.88 points in objective function). The contribution of each covariate was subsequently confirmed by stepwise backward deletion (*p* < 0.001, representing a decrease of 10.82 points in objective function). In the final model, all covariates associated with a significant increase in objective function after elimination were maintained. The choice of the model was further evaluated as described in the data analysis.

### Model validation

The internal validity of the population PK model was assessed by the bootstrap re-sampling method (repeated random sampling to produce another dataset of the same size but with a different combination of individuals) with stratification, taking into account the number of individuals at delivery and postpartum. Parameters obtained with the bootstrap replicates (250 times) were compared to the estimates obtained from the original dataset.

### Simulations

Simulations were performed for women at delivery, women 10–15 weeks postpartum (early postpartum) or healthy volunteers/late postpartum upon a single iv loading dose of 2 g of paracetamol, followed by 1 g q6h for 24 h, with or without exposure to oral contraceptives in the non-pregnant women.

## Results

The pooled dataset was based on PK studies in 69 individuals. Forty-seven pregnant women were enrolled at delivery, of whom eight were enrolled again 10–15 weeks after delivery, and seven of these eight cases again 1 year after delivery. Eight healthy female volunteers (not on oral contraceptives) were recruited, and raw data on 14 healthy female volunteers on contraceptives (13 oral contraceptives, one used a levonorgestrel covered intrauterine device) were provided by the sponsor of the Gregoire study [15]. The clinical characteristics of the different cohorts and the respective number of plasma and urine observations collected are provided in Table 1. In Table 2, the PK parameter estimates, the between and within individual variability and the bootstrap analysis of the final model are provided. Estimates in the specific subgroups (at delivery, early postpartum, late postpartum, on oral contraceptives) were provided as a Factor compared to the estimates in volunteers and late postpartum cases without oral contraceptives (reference group).

**Table 1:** Clinical characteristics of the study population. Data are provided as by mean and standard deviation or incidence

	*Pregnancy and postpartum*			*Healthy volunteers*	
	at delivery	postpartum, *early*	postpartum, *late*	no oral contraceptives	contraceptives
Number of cases	47	8 (8 of 47)	7 (7 of 8)	8	14
Plasma samples, number and time	275, 0–24 h	32, 0–6 h	28, 0–6 h	32, 0–6 h	448, 0–24 h
Urine collections, number and time	78, 0–24 h	8, 0–6 h	7, 0–6 h	8, 0–6 h	n.a.
Age (years)	30.9 (5.3)	32.1 (3.9)	32.9 (4.1)	31.1 (4.3)	23.5 (4.0)
Body weight (kg)	79.7 (12.9)	68.8 (11.2)	67.1 (13.5)	63.9 (6.6)	59.8 (8.9)
Body surface area (m^2^)	1.93 (0.19)	1.79 (0.17)	1.76 (0.2)	1.74 (0.1)	1.66 (0.14)
<37 weeks, at delivery	21/47	3/8	3/7	n.a.	n.a.
37–41 weeks, at delivery	26/47	5/8	4/7	n.a.	n.a.
Oestradiol (pg.ml^−1^)	4 833 (3 555)	86 (30)	75 (65)	79 (70)	n.a.
Progesterone (ng.ml^−1^)	118 (95)	1.1 (0.55)	0.4 (0.2)	2.8 (3.9)	n.a.
Follicular/luteal phase (number, each)	n.a.	3/0	5/0	6/2	n.a.
Oral contraceptives (number/total)	n.a.	4/8	2/7	0	13/14

**Table 2:** Parameter estimates (mean (CV%)) of the final population PK model for paracetamol and its metabolites in women at delivery, early postpartum, late postpartum or healthy volunteers, with or without oral contraceptives (OC). For Cl_PG_, V_1_ and Q_1_ both the final value and the equation is presented in which values in italic represent the value for the standard population for that parameter

Parameter	Mean			Bootstrap mean
final model	(CV%)			(CV%)
*Fixed effects*				
	*At delivery*	*postpartum, early*	*postpartum, late + healthy volunteers*	
		*10 weeks later*		
CL_PG_ (L/h)			*7.33* (8.3)	2.02 (11.1)
		0.55 (18.5) × *7.33* = 4.0		7.41 (9.7)
		OC: 1.46 (12.5) × 4.0 = 5.8	OC:1.46 × *7.33* = 10.7	0.56 (19.5)
	2.03(8.8) × *7.33* = 14.9			1.48 (10.8)
CL_PS_ (L/h)	3.86 (5.5)	3.86 (5.5)		3.82 (5.6)
	Preterm = 5.61 (7.9)			5.65 (8.4)
CL_PU_ (L/h)	0.93 (6.3) + 0.0053 (28.2) × (UP-100)			0.94 (6.5)
				0.0054 (29.8)
V_1_ (L)	1.86 (6.3) × *18.5* =	*18.5* (7.9)		1.83 (6.4)
	34.4			18.5 (7.4)
V_2_ (L)	19.7 (33.6)			22.3 (37.9)
V_8_ (L)	23.9 (5.4)			23.9 (5.0)
Q (L/h)	1.29 (15.0) × (BW/70)			1.34 (14.2)
Q_1_ (L/h)	*61.1* (6.8)	0.13 (17.9) × *61.1* = 7.9	*61.1* (6.8)	61.6 (6.3)
				0.13 (19.2)
MF	4.62 (11.8)			4.73 (10.7)
*Interindividual variability*				
ω_CLpg_^2^	0.12 (23.0)			0.12 (23.2)
ω_V1_^2^	0.09 (24.1)			0.08 (24.4)
ω_CLpu_^2^	0.12 (61.6)			0.11 (58.7)
*Residual error*				
σ^2^_(P plasma)_	0.07 (12.8)			0.07 (13.8)
σ^2^_(P G)_	0.29 (48.6)			0.29 (46.0)
σ^2^_(P S)_	0.15 (26.1)			0.14 (23.5))
σ^2^_(P u)_	0.15 (20.4)			0.15 (17.9)
σ^2^_(P plasma) Gregoire [_15_]_	0.02 (21.6)			0.02 (19.4)
σ^2^_(P plasma), additive Gregoire [_15_]_	0.016 (64.4)			0.016 (61.8)
*Performance measures*				
−2LL	5286.743			5241.994 (3.8)

Values in parentheses are CV, coefficient of variation of the parameter values; OC; oral contraceptives; CL_PG_, clearance to paracetamol-glucuronide; CL_PS_, clearance to paracetamol-sulphate, CL_u_, clearance to paracetamol unchanged; UP, urine production (urine volume (ml) divided by collection time (h)); V_1_, central volume; Q and Q_1_, intercompartmental clearance between central and peripheral volumes; BW, body weight; V_2_ and V_8_, peripheral volumes; MF multiplication factor for k17 compared with k35 and K46; ω^2^ variance, the square root of the exponential variance of η minus 1 is the percentage of interindividual variability in the parameters; σ^2^ proportional within individual variance; −2LL, objective function

**Fig. 2:**
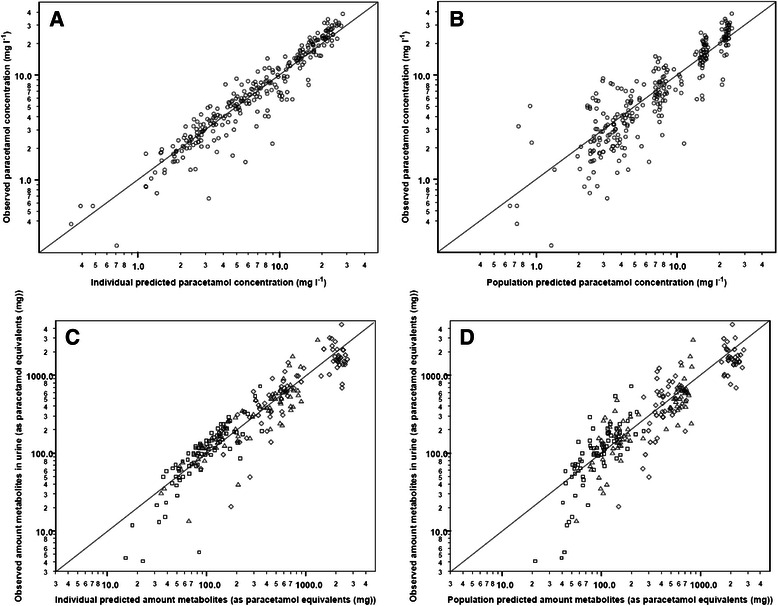
Diagnostics plots for the final PK model for **a** women at delivery, **b** women in early postpartum and **c** women in late postpartum and **d** healthy volunteers including observations vs individual predictions (*left*) and observations vs population predictions (*right*) for paracetamol concentrations in plasma (*circle, upper panels*) and amount of paracetamol-glucuronide (*diamond*), paracetamol-sulphate (*triangle*), and unchanged paracetamol (*square*) in urine (*lower panels*) as paracetamol equivalents with x = y identity line. The solid symbols indicate women on contraceptives, the open symbols women with no contraceptives. In panel **c** the group of healthy volunteers on contraceptives (*n* = 14, Gregoire) are distinguished from women in late postpartum by a *triangle down*, the healthy volunteers with no contraceptives (*n* = 8) by symbols plus (*circle plus; diamond plus, triangle box* and *square plus*)

Figure 2 shows the *observed* versus *individual predicted* concentrations/amounts and the *observed* versus *population predicted* concentrations/amounts for plasma and urine observations for the final model for (Fig. 2a) women at delivery, (Fig. 2b) women in early postpartum (10–15 weeks after delivery), and (Fig. 2c) late postpartum (1 year) or healthy volunteers, with or without oral contraceptives.

The systematic covariate analysis showed that being at delivery was the most significant covariate for clearance to paracetamol glucuronide (ΔOF 78.9, Factor = 2.03). The influence of oestradiol levels or progesterone levels on glucuronidation clearance - implemented as a power function - resulted in decreases in objective function of 60.5 points and 68.9 points respectively. However, implementation of oestradiol or progesterone in addition to being at delivery on glucuronidation did not further improve the model. Women in early postpartum showed a decreased paracetamol glucuronidation clearance (Factor = 0.55) compared to healthy women (ΔOF = 26.4, *vs* basic model; ΔOF 29.1 backward deletion *vs* final model, *p* < 0.001). Women taking oral contraceptives showed increased paracetamol glucuronidation clearance versus women without oral contraceptives (Factor = 1.46, ΔOF 6.9, *P* < 0.01, *vs* basic model; ΔOF = 15.4 backward deletion *vs* final model). Being at delivery did not prove to be a significant covariate for clearance to paracetamol sulphate. However, clearance to paracetamol sulphate was higher in pregnant women who delivered preterm (<37 weeks, Factor = 1.34) compared to term delivery and non-pregnant women. Finally, clearance of unchanged paracetamol was dependent on urine flow rate (diuresis, mean urine flow 100 ml/h). The addition of urine production (urine volume, ml divided by collection time, h) as a linear equation on clearance of unchanged paracetamol for the measured range 20–283 ml/h resulted in a significant decrease of objective function compared with the basic model (ΔOF 38.6, *P* < 0.001). For missing values the urine production was assumed to be 100 ml.h^−1^.

**Fig. 3:**
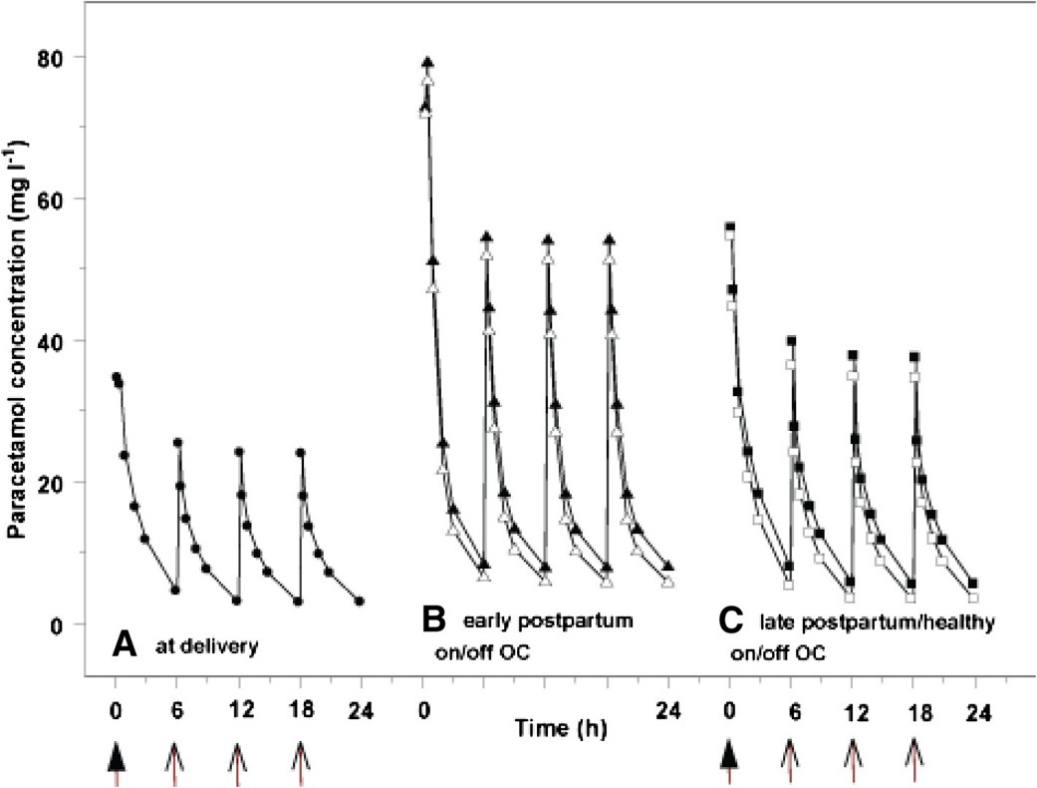
Model based simulation of plasma paracetamol disposition after 2 g loading dose, followed by 1 g paracetamol every 6 h in women with different clinical characteristics [at delivery (**a**, *circle*), in early postpartum (**b**, *triangle*), in late postpartum or in healthy volunteers (**c**, *cube*)]. For the **b** and **c** panel, simulations are provided with (*white*) or without (*black*) exposure to oral contraceptives

Central volume standardized for body weight significantly improved the model. However, being at delivery as a covariate for the central volume proved to be more significant. Addition of body weight on central volume for the different groups did not further improve the model. The inter-compartmental clearance (Q) standardized for body weight (BW) proved significant. The inter-compartmental clearance Q1 was reduced in women in early postpartum (Factor = 0.13) relative to the population mean of 61.1. (ΔOF = 46.6 backward deletion *vs* final model). The group late postpartum could not be identified as significant covariate, which would suggest that the pharmacokinetics 1 year postpartum equals the healthy volunteer group. The impact of these covariates (pregnancy, early/late postpartum, volunteers, with or without oral contraceptives) on plasma paracetamol disposition is illustrated in Fig. 3.

## Discussion

**Fig. 4:**
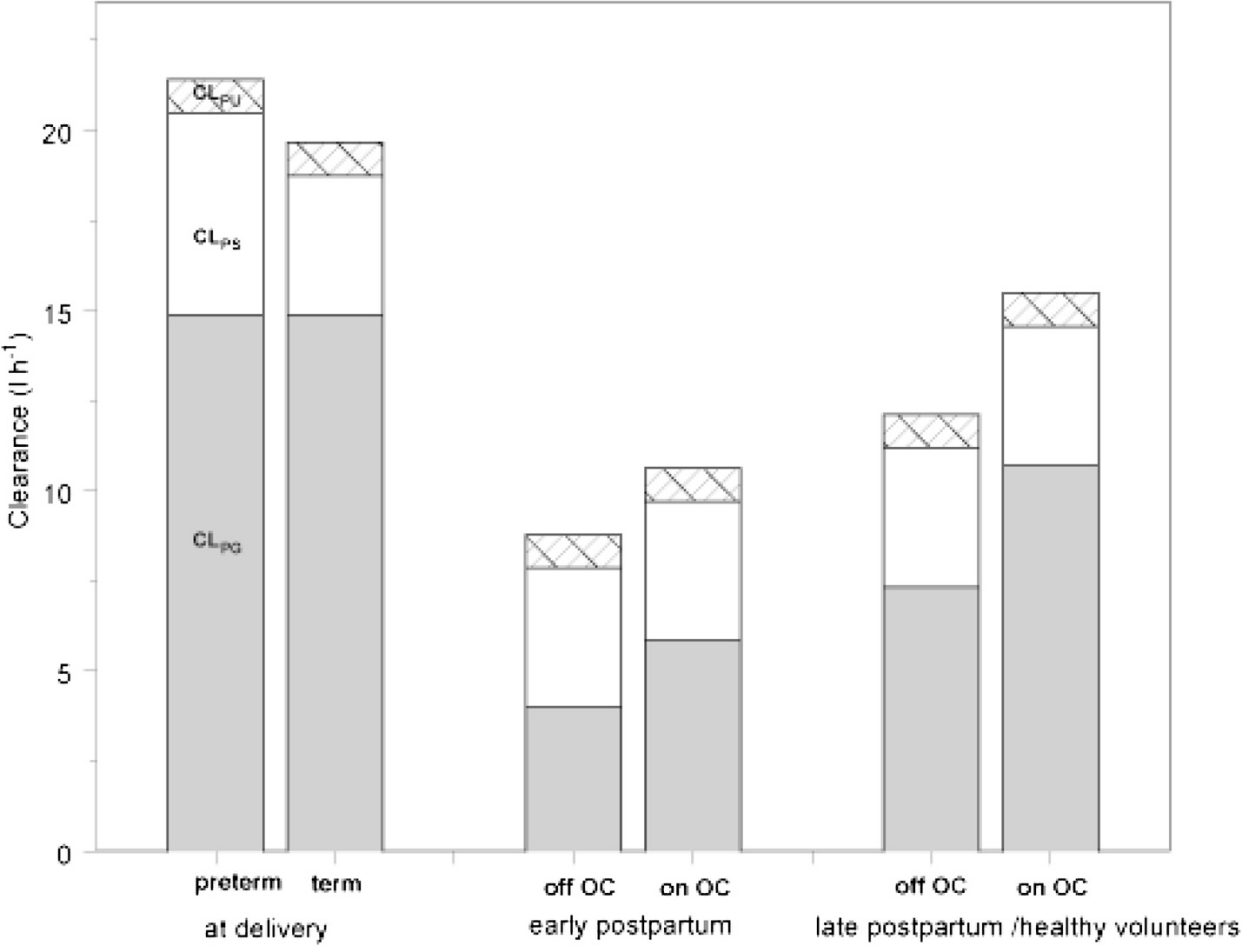
Clearance to paracetamol-glucuronide (CL_P-G_, *grey*, l.h^−1^), clearance to paracetamol-sulphate (CL_P-S_, *transparent*, l.h^−1^) and clearance of unchanged paracetamol (CL_P-U_, *striped*, l.h^−1^) as estimated at delivery, in early postpartum, or in late postpartum/healthy volunteers are provided with the impact of the other covariates (preterm on CL_P-S_ at delivery, oral contraceptives (OC) on CL_P-G_ in non-pregnant women). The sum reflects the total paracetamol clearance, while the coefficients of variation can be retrieved in Table 2

The current study explored the variability in the different metabolic and elimination clearance estimates in young women following iv paracetamol administration. To allow for an analysis of the different metabolic pathways, we applied an earlier described model, based on simultaneous collection of plasma and urine [16]. Using this approach, we clearly confirmed the significantly higher (Factor = 2.03, 15.8 l.h^−1^) clearance to paracetamol glucuronide at delivery and significantly lower (Factor = 0.55, 4.66 l.h^−1^) clearance in early postpartum when compared to healthy female volunteers (7.33 l.h^−1^) [11, 16]. In addition, the use of oral contraceptives (Factor = 1.46) – obviously limited to non-pregnant women - was also found to affect clearance to paracetamol glucuronide. Besides these major effects on paracetamol metabolic clearance, there was a minor impact of preterm (Factor = 1.34), but not for term delivery on clearance to paracetamol sulphate and of the urine flow on elimination of unchanged paracetamol in urine (Fig. 4). Finally, these clinical covariates performed better as predictors of altered paracetamol glucuronidation clearance when compared to oestradiol or progesterone levels.

Both the impact of pregnancy and oral contraceptives on intravenous paracetamol clearance have been reported earlier in literature (Table 3) [9, 10, 14, 16, 18, 19]. In the current pooled analysis, we clearly linked this raised clearance with a raised paracetamol glucuronidation activity and initially hypothesized that this was associated with oestradiol as biomarker. This hypothesis was based on the fact that endogenous estrogens are both a substrate as well as an inducer of glucuronidation enzymes [12, 13, 20, 21], and similar observations have been described for ethinyloestradiol [22]. However, the use of oestradiol as biomarker in itself was not superior to the use of the more readily available clinical characteristics (pregnancy, postpartum, exposure to oral contraceptives) in our model.

**Table 3:** Overview of the pharmacokinetics of intravenous (iv) paracetamol in cohorts of women as retrieved in literature

Author	Study characteristics		Clearance (l/h)	Distribution volume (l/kg)
Ochs et al. [18]	single iv, 650 mg, young women, age matched study design			
	Controls (*n* = 10), 21–30 year, 54 (SE 2.1) kg		16.8 (SE 0.6)	0.98 (SE 0.08)
	Oral contraceptives (*n* = 10), 62 (SE 2.5) kg		22.7 (SE 2.3)	0.98 (SE 0.06)
Sonne et al. [19]	single iv, 1 000 mg, 2 episodes in each individual		16.6	1.01
	3 women 54–56 kg, 29–33 years			
Scaveno et al. [14]	single iv, 650 mg, 30 post-menopausal women			
	controls (*n* = 18): 45 (SE 3.9) years, 64.9 (SE 3.3) kg		16.6 (SE 0.69)	0.85 (SE 0.04)
	conjugated oestrogens (*n* = 12): 46 (3.4) years, 60.2 (1.7) kg		16.6 (SE 0.25)	0.82 (SE 0.05)
Abernethy et al. [9]	single iv, 650 mg, 16 women			
	controls (*n* = 8): 23–32 years, 48–66 kg		13.7 (SD 1.26)	0.96 (SD 0.08)
	oral contraceptives (*n* = 8): 21–36 years, 48–77 kg		20.0 (SD 1.68)	1.04 (SD 0.08)
Wynne et al.[10]	single iv, 500 mg, 42 female/5 male volunteers, *.*all results pooled			
	healthy, young:	25 (SE 1) years, 59 (SE 2) kg	16.6 (SE 0.71)	1.00 (SE 0.04)
	healthy, elderly:	73 (SE 1) years, 66 (SE 2) kg	14.6 (SE 0.79)	1.07 (SE 0.03)
	frail, elderly:	82 (SE 2) years, 53 (SE 4) kg	7.9 (SE 0.32)	0.81 (SE 0.03)
Kulo et al. [16]	single iv, 2 000 mg			
	28 cases, at delivery	31.5 (20–42) years, 79 (57–110) kg	20.3 (11.8–62.8)	0.72 (0.52–1.56)

*SE* standard error, *SD* standard deviation

Besides the impact of pregnancy and oral contraceptives on paracetamol glucuronidation, the clearance of unchanged paracetamol was dependent on the urine output. This confirms earlier observations of Miners et al., who quantified the effects of high and low urine flow rates on the urinary metabolic ratios for paracetamol glucuronidation, sulphation and oxidation at steady-state in seven (four female, three male) healthy young adults [7]. Metabolic partial clearances were unaffected by urine flow rate, but individual paracetamol metabolic ratios varied 2.5- to 3.2-fold over a 7.4-fold range of urine flow rates (48–360 ml.h^−1^).

Beyond changes in paracetamol disposition, we hypothesize that this pattern of raised phenotypic glucuronidation driven by pregnancy or oral contraceptives is of relevance to explain and predict within and between individual variability in disposition of drugs that mainly undergo UDP-glucuronosyltransferase (UGT)1A6, 1A1, 1A9 or 2B15 driven glucuronidation. Consequently, we anticipate a similar pattern for other drugs that undergo glucuronidation, including lamotrigine (UGT1A4, plasma concentrations increase in postpartum, range + 75–351 %, reflecting decreased clearance), propofol (UGT1A9, clearance 35 % higher during pregnancy) or benzodiazepines (UGT2B7/15, clearance 75 % higher during pregnancy) [4, 12, 20–26]. Similar to the development and validation of model-based approaches in the field of maturation based on system specific information [27, 28], the quantitative functions described can be used to quantify the impact of pregnancy or oral contraceptives on phenotypic UGT1A1 or UGT1A6 glucuronidation.

## Conclusions

Variability in paracetamol glucuronidation elimination in young women was in part explained by pregnancy, early postpartum or exposure to oral contraceptives. Oestradiol or progesterone plasma levels also explained increased paracetamol glucuronidation elimination. However, implementation of oestradiol or progesterone in addition to being at delivery did not further improve the model. We hypothesize that the pattern of raised phenotypic glucuronidation and its variability in young women is of relevance to predict within and between individual variability in disposition of any drug that is subject to glucuronidation.

## Abbreviations

BW: Body weight
CL: Clearance
CYP: Cytochrome p450
CV: Coefficient of variation
ELISA: Enzyme-linked immunosorbent assay
FDA: Food and drug agency
GA: Gestational age
GCP: Good clinical practice
HPLC: High performance liquid chromatography
iv: Intravenous
k: Elimination rate
MF: Multiplication factor
NAPQI: N-acetyl-p-benzoquinone-imine
OC: Oral contraceptives
PD: Pharmacodynamics
P-G: Paracetamol glucuronide
PK: Pharmacokinetics
P-O: Paracetamol, oxidative metabolites
P-S: Paracetamol sulphate
P-U: Unchanged paracetamol
Q: Intercompartmental clearance
V: Distribution volume
